# Nursing Process applicability in women’s care in Primary Health Care

**DOI:** 10.1590/0034-7167-2025-0501

**Published:** 2026-08-21

**Authors:** Jéssica Kelly Ramos Cordeiro, Marquiony Marques dos Santos, Ana Luisa Brandão de Carvalho Lira, Taís Feitosa da Silva, Jairo Porto Alves, Nayara Vilela de Farias Serranegra, Manoel Vieira de Miranda, Claudia Santos Martiniano

**Affiliations:** I Universidade de Pernambuco. Recife, Pernambuco, Brazil; II Universidade do Estado do Rio Grande do Norte. Caicó, Rio Grande do Norte, Brazil; III Universidade Federal do Ceará. Fortaleza, Ceará, Brazil; IV Universidade Estadual da Paraíba. Campina Grande, Paraíba, Brazil; V Faculdade Israelita de Ciências da Saúde Albert Einstein. São Paulo, São Paulo, Brazil; VI Universidade de São Paulo. São Paulo, São Paulo, Brazil

**Keywords:** Primary Health Care, Nurse’s Role, Nursing Process, Women’s Health, Nursing Care., Atención Primaria de Salud, Rol de la Enfermera, Proceso de Enfermería, Salud de la Mujer, Atención de Enfermería.

## Abstract

**Objectives:**

to analyze the application of the Nursing Process stages in women’s care in Primary Health Care.

**Methods:**

a cross-sectional study was conducted from May to July 2022 in 17 Basic Health Units across four Brazilian municipalities, involving 25 nurses and 92 medical records. A questionnaire was administered to nurses, and data analysis was performed using descriptive statistics with SPSS^®^ software version 25.0. Medical records were read and analyzed.

**Results:**

the execution of the Nursing Process was partial, and all phases had incomplete records. Assessment and planning were the most prevalent, while nursing diagnosis, implementation, and assessment were the weakest.

**Conclusions:**

incomplete Nursing Process records compromise the documentation of nursing work regarding patient care, potentially leading to ethical and legal implications for professionals. It is necessary to invest in training and foster professional interest in the Nursing Process application and recording.

## RESUMEN

**Objetivos:**

analizar la aplicación de los pasos del Proceso de Enfermería en la atención a mujeres en Atención Primaria de Salud.

**Métodos:**

estudio transversal, de mayo a julio de 2022, en 17 Unidades Básicas de cuatro municipios brasileños, con la participación de 25 enfermeras y 92 historias clínicas. Se administró un cuestionario a las enfermeras, y se realizó un análisis de datos mediante estadística descriptiva con el programa SPSS^®^, versión 25.0. Se analizaron las historias clínicas.

**Resultados:**

la ejecución del Proceso de Enfermería fue parcial y todas las fases presentaron registros incompletos. La evaluación y planificación fueron las más frecuentes, mientras que el diagnóstico, la implementación y evaluación de enfermería fueron las más deficientes.

**Conclusiones:**

los registros incompletos del Proceso de Enfermería comprometen la documentación del trabajo de enfermería en relación con la atención al paciente, lo que podría conllevar implicaciones éticas y legales para los profesionales. Es necesario invertir en formación y fomentar el interés profesional en la aplicación y el registro del Proceso de Enfermería.

## INTRODUCTION

In Brazil, the role of nurses in Primary Health Care (PHC) is becoming established as an instrument for change in healthcare practices within the Brazilian Health System (In Portuguese, *Sistema Único de Saúde* - SUS). These professionals’ technocratic perspective has been replaced by a broader vision of care, centered on comprehensiveness, intervention in risk factors, disease prevention, and health promotion^(1)^. In this context, the Nursing Process (NP) acts as a tool that enables quality care. In this way, nurses have the opportunity to strengthen their profession and consolidate their professional space within PHC^(2)^.

Within the context of care, nursing work processes are complex and multifaceted, classically unfolding into five processes: assist; administer; teach; research; and participate politically^(3)^. To perform their work efficiently, nurses need to master several skills, working in two main dimensions: caregiving and management^(4)^. The care dimension focuses on direct care for individuals, families, and communities, while the management dimension focuses on coordinating resources and the care providers themselves to make care possible. Therefore, it is expected that nurses in PHC will coordinate not only clinical-care practices, such as appointments and ordering tests, but also team management and monitoring of the population’s health status^(3,5)^.

To organize and improve quality of care, the NP is the main methodological tool, allowing nurses to make decisions and plan actions for promotion, prevention, and intervention^(3,6)^. Thus, the NP application transcends the mere formality of registration; it is the operationalization of the “care work process”^(3)^, grounding clinical reasoning and professional autonomy in comprehensive care planning in PHC^(6)^.

In the Brazilian context, the Federal Nursing Council^(7)^, through Resolution 736/2024, provides for the implementation of the NP in all socio-environmental contexts where nursing care occurs, organizing itself into five interrelated, interdependent, recurrent and cyclical stages: nursing assessment; nursing diagnosis (ND); nursing planning; nursing implementation; and nursing evolution^(7)^.

The NP is a scientific method used to plan, execute, and assess nurses’ work^(8)^. This is an essential component of legislation that provides legal support for nursing, outlining nurses’ exclusive activities. Its implementation favors the work of this professional category, establishing patient care in sequential stages, with interventions based on scientific evidence and putting logical reasoning into practice^(6)^.

To support the NP, classification systems are used, the most widely used being the NANDA International Taxonomy (NANDA-I)^(9)^, the Nursing Interventions Classification^(10)^, the Nursing Outcomes Classification^(11)^, the International Classification for Nursing Practice^(12)^ and the International Classification of Public Health Nursing Practices^(13)^.

It is noteworthy that nurses’ work, as a strengthening agent of PHC, should provide quality care to the population through nursing appointments (NAs) and based on classification systems, with efficient actions accepted by the community^(14)^. Therefore, it is essential that nurses continuously seek improvement, aiming at the appropriate determination of interventions in NAs and recognizing that the NP is continuous and permanent, which positively reflects on quality of care^(15)^. It is known that its use is mandatory in all scenarios where this professional works with individuals, families, and communities. In this regard, PHC stands out as an important space for this implementation^(7,16,17)^.

Although some studies discuss the NP applicability in PHC^(8,6,13,18)^, they do not encompass the distinct regions of Brazil in a single study, nor are they related to women’s health in its entirety. In this regard, considering that women’s health is the area that grants the greatest professional autonomy to nurses, due to the provision of care involving Pap smear collection, prenatal and postpartum care, gynecological complaints, acute events, family planning, ordering of tests, and prescription of medications^(19)^, among other activities that are characterized as advanced nursing practices^(20)^, this study poses the following question: do nurses working in PHC apply the NP during NAs in women’s health?

## OBJECTIVES

To analyze the application of the NP stages in women’s healthcare in PHC.

## METHODS

### Ethical aspects

The study was conducted in accordance with national and international ethical guidelines. It was approved by the *Hospital Israelita Albert Einstein - São Paulo* Research Ethics Committee, whose opinion is attached to this submission. Hence, this study meets the requirements set forth in Resolution 466/2012^(21)^ from the Brazilian National Health Council, which regulates research involving human beings.

### Study design, period and location

This is an observational, multicenter, exploratory, cross-sectional study with a quantitative approach, guided by the STrengthening the Reporting of Observational studies in Epidemiology tool. It was conducted in 17 Basic Health Units (BHUs) located in four Brazilian municipalities, namely Parelhas (Rio Grande do Norte), São Paulo (São Paulo), Manaus (Amazonas), and Carneiros (Alagoas), encompassing northeastern, northern, and southeastern Brazil. The municipalities were chosen for convenience, as the researchers reside in these states, facilitating data collection. BHU selection was also for convenience, following invitations from the researchers and acceptance by the nurses working in these settings. The sample included nurses working in PHC, all actively performing their duties on the day of data collection, totaling 25 participants. Nurse managers were excluded from the study.

### Study protocol

Data collection took place between May and July 2022. At that time, the researchers approached and invited participants, explaining the research objectives, methodology, risks, and benefits. Upon acceptance, participants signed the Informed Consent Form and the Informed Assent Form when necessary. Subsequently, a specific characterization questionnaire for this population group was applied using the Research Electronic Data Capture (REDCap) platform, in addition to an Authorization Form for the Use of Image and Voice.

Subsequently, medical records were analyzed, and the records of the care provided by nurses were accessed, with the aim of identifying compliance with the NP stages in the appointments. Data were collected through copies of the progress notes in physical or electronic medical records, according to what the unit had available, provided by the participating nurses and archived in an institutional drive, being analyzed after data collection completion, between August and September 2022. In total, 92 medical records were analyzed.

The data collection instrument is a checklist developed by the researchers. For this purpose, the stages were adapted to the Subjective, Objective, Assessment, and Plan (SOAP) method, considering that this is the record format available in the national e-SUS medical record^(22)^, and the application was carried out through the REDCap platform (Chart 1).

**Chart 1:** Nursing Process assessment instrument using the Subjective, Objective, Assessment, and Planning method

NURSING PROCESS STAGES	ANALYSIS
Data collection	
Subjective data	Did the nurse seek to understand the demand from the user’s point of view? Did the quality of the process involve individual and global assessment to identify relevant nursing phenomena?
Objective data	
Assessment	
Physical examination	Does it include the user’s needs, problems, and personal resources? Does it include the reason that led the user to seek nursing appointment? Were social, gender, spiritual, and physiological aspects described?
Nursing diagnosis	
Did they provide a nursing diagnosis?	Are the nursing diagnoses recorded? Are the nursing diagnoses numbered in order to be monitored in the nursing records? Do the nursing diagnoses contain etiologies related to the corresponding signs and symptoms? Are the nursing diagnoses identified recorded according to the taxonomy used by the institution? If not, which one did the nurse use?
Does the nursing diagnosis correlate with the data collected?	
Did you use ICPC?	
Does the ICPC correlate with the data collection presented?	
Planning	
Goals/Expected Results	Is the nursing plan documented? Is standardized language used? Is the nursing plan developed according to: how will it be carried out? How often? By whom will it be carried out? Is the nursing plan documented according to the problems or diagnoses identified?

*Note: ICPC - International Classification of Primary Care.*

*Source: Cordeiro^(20)^; Barreto^(23)^; Carrer^(24)^; Serranegra^(25)^; Reis^(26)^; Almeida^(27)^ - Recife, Pernambuco, Brazil, 2025.*

### Analysis of results and statistics

During record analysis, the following values were assigned: 0 = not applicable; 1 = absent (when there is no record of each sub-item in the medical record); 2 = partially present (when there is a record of only one aspect); and 3 = present (when the record is complete).

Data were transferred to a spreadsheet and the variables were analyzed using descriptive statistics (frequency and percentage) with the Statistical Package for the Social Sciences software version 25.0. Data assessment was performed, considering completeness, illogical values, and data entry. The mean and standard deviation of the appointment time and age variables were calculated.

## RESULTS

Twenty-five nurses participated in this study, predominantly female (80%), with a mean age of 39.13 ± 7.36 years. The majority identified as brown (60%), gaining above seven minimum wages (56%) and residing in the city of Manaus (40%). In the descriptive analysis of academic background, most respondents reported having received classes on NP during their undergraduate studies (88%), and 100% stated they had graduate training, with 44% of this training focused on specialization in family and community health, collective health, or public health. Most also participated in courses in the area of Women’s Health in the last year (64%).

Concerning professional experience, a significant portion of nurses stated they had between six and ten years of experience (48%), and one to five years of experience in the Family Health Strategy (36%), which was the type of health unit they were working in during the research (64%).

Table 1 presents the distribution of contextual variables in absolute and relative values for the nurses participating in the research. According to the table, regarding working conditions, most nurses had a commute time of up to 30 minutes to arrive at work, worked 40 hours per week, used the NANDA International taxonomy to develop NDs, and applied Wanda Horta’s Theory of Human Needs. Most did not have another employment relationship, had a multidisciplinary team integrated into the health team, worked with electronic medical records, and had a dedicated room for NA. A considerable portion used nursing protocols in their work routine (56%), with the most cited being the Primary Care Booklets and the Ministry of Health protocols. Regarding motivation for nursing, a significant portion expressed satisfaction (52%) (Table 1).

**Table 1:** Distribution of contextual variables in absolute and relative values of the nurses participating in the research (N=25), Recife, Pernambuco, Brazil, 2025

Variables	Categories	n	%
Time spent commuting to work	Up to 30 minutes	12	48.0
	Between 31 and 60 minutes	8	32.0
	Between 61 and 120 minutes	4	16.0
	More than 121 minutes	1	4.0
Weekly working hours	30 hours per week	1	4.0
	40 hours per week	22	88.0
	Other	2	8.0
	NANDA-I	8	32.0
Which nursing diagnosis taxonomy do you use in your daily work?	None	8	32.0
	International Classification for Nursing Practice	6	24.0
	Other	3	12.0
Which nursing theory do you use in your daily work?	Human Needs Theory - Wanda Horta	13	52.0
	Does not use nursing theory	7	28.0
	Other	3	12.0
	Environmental THEORY - Florence Nightingale	1	4.0
	Self-Care Theory - Dorothea Orem	1	4.0
Do you have any other employment outside of Primary Health Care?	No	15	60.0
	Yes	10	40.0
Do you have a multidisciplinary team linked to your healthcare team?	Yes	16	64.0
	No	9	36.0
Does your unit have an electronic medical record system?	Yes	22	88.0
	No	3	12.0
Does your unit have a room for nursing appointments?	Yes	18	72.0
	No	7	28.0
Are nursing protocols followed in your unit?	Yes	14	56.0
	No	11	44.0
	Primary Care Handbook from the Ministry of Health	20	80.0
	Ministry of Health Protocols	18	72.0
	Protocols from their municipal health department	11	44.0
	Regional Nursing Council protocol	7	28.0
	State protocol	3	12.0
	Protocol of another municipality	2	8.0
How motivated do you feel to perform your professional duties?	Very satisfied	5	20.0
	Satisfied	13	52.0
	Somewhat satisfied	3	12.0
	Neither satisfied nor dissatisfied	1	4.0
	Dissatisfied	3	12.0

*Note: conventional symbols used: N - number of participants; % - representative percentage.*

Regarding the type of NA, 28% of users sought prenatal care, followed by 26% who sought a Pap smear and 10% who came to present test results. Among the main symptoms reported by patients during data collection, a significant portion mentioned difficulty sleeping (26%), dizziness (22%), weakness (20%), episodes of crying or sadness (16%), and shortness of breath (13%).

The mean appointment time was 22.88 ± 17.17 minutes, with the majority of appointments lasting ≤ 20 minutes (55%). In some cases, patients were accompanied during their appointment (20%), with the accompanying persons being children (6%) and siblings (6%). In 42% of NAs, there were interruptions, and in 64%, nurses sought a physician for interappointment. Among the appointments, 32% were return visits or longitudinal follow-up, while in 67%, nurses mentioned the need for a new appointment. The NAs were recorded in 92% of medical records, with 73% in electronic medical records and in SOAP format.

In relation to the NP stages, nursing assessment was mostly recorded in a partial manner, with a predominance of subjective data (49%), objective data (74%), and physical examination data (41%). NDs were absent in 71% of the analyzed records, and when present, only 9% were related to data collection. The record format was based on the International Classification of Primary Care (ICPC) (74%), and 62% were present in relation to the ICPC and data collection.

As for the planning stage, it was found that 14% of records presented a partial description of the goals and expected results, and 4% showed a relationship with the diagnosis. Furthermore, the plan prescription stage accounted for 61% of records in medical charts, with 11% of these prescriptions applied to the expected results and 10% to the diagnosis, both partially. The implementation stage was present in only 5%, and the plan’s progress was evidenced in 12% of medical records.

## DISCUSSION

This study showed that nurses performed NAs in an incipient and weak manner and applied the NP stages partially, with incomplete record-keeping. Nursing assessment and planning were the most frequently used, while implementation and plan development were the most incipient. Studies have presented similar results, referring to fragmentation when carrying out NAs, fragility in record-keeping^(28-30)^ and partial development of the NP stages in PHC^(23-27)^.

The NP fragmentation is frequently observed in the completion of medical records in PHC, and several factors have been identified that hinder the NP applicability, the most cited in the literature being: lack of training for the NP application and of physical spaces to carry out NAs; work overload; professional devaluation^(6,31,32)^; inadequate team size; lack of institutional and multidisciplinary team support; interruptions during NAs; limitations regarding scientific knowledge; perception of wasted time in the NP execution^(29,32)^; and lack of protocols to support its applicability in practice^(8,33)^.

Nurses understand that knowledge about the NP is essential for their implementation and documentation. However, at the same time, they consider this knowledge insufficient from their academic training, resulting in inadequate and partial use of this technology^(18,32)^. Although most participants in this study received instruction on the NP during their undergraduate studies, traces of this training remain, highlighting its limitations in PHC practice. Understanding this reality is essential for developing innovative practices through initiatives that combine teaching and professional practice^(34)^.

The lack of support from oversight bodies and managers in PHC has also been identified as a frequent weakness that negatively impacts the NP implementation^(29,30,32)^. Therefore, the importance of implementing strategies that ensure the effective operationalization of the NP in healthcare services is reinforced, considering the specificities of technological transformations, conceptual advancements, and the development of nursing practice and regulation in Brazil^(35,36)^.

From this perspective, the role of universities in this scenario is to mediate and focus on strengthening the theoretical foundations related to the NP, contributing to clinical reasoning and highlighting that the NA in women’s health is not only related to gynecological complaints, but to the integrality of care, considering biopsychosocial, cultural, and individual aspects. Therefore, nurses need to provide quality care based on the best scientific evidence, applying the NP in all appointments^(23)^.

Therefore, there is a need to reorient nursing school curricula, in which content related to the NP in PHC is included and offered in a specific and coordinated manner. Its use in academic training provides students with an understanding of professional legislation, fostering a broader view of nursing actions^(27)^. Concomitantly, local managers and higher authorities need to raise awareness, strengthening and promoting continuing education workshops on the NP and the expansion of the scope of nursing, enabling nurses to incorporate the expected competencies during appointments^(23)^.

Furthermore, it is worth highlighting the role of the nurse as an active participant in this process. It is essential that this professional actively seeks knowledge to qualify and improve the care provided to patients, as well as perform tasks within their autonomy and competence.

It is understood that autonomy is related to professional knowledge, since nurses who are trained demonstrate better mastery of their work, contributing to the provision of qualified care and gaining visibility that, consequently, will reflect in the autonomy achieved^(37)^. From this perspective, in order to provide autonomous care, it is necessary to recognize the specific knowledge of nursing. Failures in this process hinder clinical reasoning, potentially turning the NP into a mere execution of care tasks, thus disqualifying it as a scientific method^(38)^.

In this study, nursing assessment and plan prescription were the stages most frequently recorded by nurses, supporting other research^(28,34)^. This can be justified by the fact that nurses have access to manuals, Primary Care Booklets, and ministerial and institutional protocols that support their professional practice, contributing to the autonomous exercise of nursing activities^(19)^. The use of these documents can significantly contribute to implementing NP in PHC^(6)^.

In contrast, implementation and evolution were shown to be the most incipient stages, weakening the NP comprehensiveness and completeness by the professional who prescribed the care. According to Faruch *et al*.^(39)^ the NP implementation aims to guarantee, based on scientific evidence, the best conduct and quality of care provided to patients. This contributes to the continuous development of the theoretical, practical and interactive skills of healthcare professionals.

When nurses incorporate the NP into their practice, they ensure that their activities are carried out efficiently and based on scientific evidence. This provides them and their entire multidisciplinary team with the necessary security for performing procedures. In this way, resources are offered for planning care in response to nursing prescriptions, organizing and guiding the team regarding care priorities, which favors a comprehensive and holistic approach to patients^(40)^.

Regarding the nursing theory used in their work environment, participants reported using Wanda Horta’s Theory of Basic Human Needs as a framework. This theory, a pioneer of the NP in Brazil, guides nursing assessment and supports the recording of subsequent stages of the process. This theory identifies the NA as essential for improving quality of care, making it more humanized and allowing nurses to act with greater autonomy and independence in their interactions with patients^(8)^.

It is worth noting that the complete development of the NP must be supported by a nursing theory, associated with proper documentation in patients’ medical records^(41)^, since, when well documented, it allows for patient-centered care and reflects the quality of care provided^(42)^.

The apparent contradiction between the high level of autonomy perceived by nurses in women’s healthcare^(43)^ and the only partial application of the NP, reveals the complexity of the work process in PHC, which encompasses not only “assisting”, but also “administering”, “teaching”, and “participating politically”^(3)^. It is therefore suggested that the reported autonomy may be exercised more in the execution of ministerial protocols, such as prenatal care^(5)^ and cancer screening^(43)^, than in the full application of the NP.

This study supports that women’s healthcare, although a priority on the agenda, focuses intensely on the reproductive cycle, neglecting areas such as menopause or addressing violence^(43)^. The weaknesses found in the “implementation” and “evolution” NP stages are critical, as they ensure continuity and reassessment of care. Partial adoption transforms the NP into a fragmented and bureaucratic act, limiting its potential. This is particularly relevant because evidence indicates that it is the quality of the care process (which the NP should guarantee) that positively impacts health outcomes, even minimizing the effects of socioeconomic inequities^(44)^.

Therefore, the failure to fully apply the NP, even in a scenario of autonomy, represents an underutilization of that same professional autonomy, which should be the cornerstone for qualification of care. Thus, it is essential not to normalize the partial use of the NP, especially in women’s health. Furthermore, by abandoning the use of the NP, nursing does a disservice to its own profession, relegating to a secondary position the body of knowledge that provides scientific grounding, contributes to nursing evolution, and guarantees the power to decide on its conduct in patient care^(45)^.

### Study limitations

The limitation of this study may be related to the sample size. However, since different regions of Brazil were studied, the heterogeneity of the sample was maintained across the researched scenarios. It is noteworthy that this research made it possible to understand the importance and necessity of developing NAs in a systematic and effective way and the NP applicability in its entirety.

### Contributions to health, nursing, or public policy

The adoption of specific tools for nurses’ work improves professional work quality, strengthens systematized care, structures work processes and routines, and facilitates information recording and organization^(23,24,32)^. In light of the above, the need for nurses to empower themselves in matters that are exclusive to them is reaffirmed, seeking knowledge to increase professional recognition and implement evidence-based interventions^(46,47)^. This study contributes to nurses’ reflection on the importance of implementing the NP as a strategy for managing care and consolidating professional autonomy.

## CONCLUSIONS

Incomplete NP records compromise the documentation of nursing work regarding patients’ clinical condition, potentially leading to ethical and legal implications for professionals. In this regard, investment in training and ongoing education on the NP in women’s health appointments, specifically for nurses working in PHC, is essential, as is fostering interest among these professionals in applying it in their clinical practice.

Failure to carry out the np, in accordance with the precepts established by Resolution 736/2024^(7)^, may cause adverse impacts on quality of care, including the lack of systematic patient assessment, compromising continuity of care, early identification of complications and personalization of interventions, which results in greater risk to patient safety and undesirable clinical outcomes.

This study promotes reflection on the roles of nursing professionals and envisions the potential of NA as a methodological tool to systematize care and strengthen PHC.

## Data Availability

The research data are available only upon request.
