# Supplementary material for: Nursing Process applicability in women’s care in Primary Health Care
Source: Rev Bras Enferm. 2026 Aug 21;79:e20250501. doi: 10.1590/0034-7167-2025-0501 (PMC13505561; doi:10.1590/0034-7167-2025-0501)
Supplement: Translation

# Translation: Aplicabilidade do Processo de Enfermagem no cuidado à mulher na Atenção Primária à Saúde

**DOI:** 10.1590/0034-7167-2025-0501pt

**Authors:** Jéssica Kelly Ramos Cordeiro, Marquiony Marques dos Santos, Ana Luisa Brandão de Carvalho Lira, Taís Feitosa da Silva, Jairo Porto Alves, Nayara Vilela de Farias Serranegra, Manoel Vieira de Miranda, Claudia Santos Martiniano

**Affiliations:** I Universidade de Pernambuco. Recife, Pernambuco, Brasil; II Universidade do Estado do Rio Grande do Norte. Caicó, Rio Grande do Norte, Brasil; III Universidade Federal do Ceará. Fortaleza, Ceará, Brasil; IV Universidade Estadual da Paraíba. Campina Grande, Paraíba, Brasil; V Faculdade Israelita de Ciências da Saúde Albert Einstein. São Paulo, São Paulo, Brasil; VI Universidade de São Paulo. São Paulo, São Paulo, Brasil

**Keywords:** Atenção Primária à, Saúde, Papel do Profissional de Enfermagem, Processo de Enfermagem, Saúde da Mulher, Cuidados de Enfermagem.

## Abstract

**Objetivos:**

analisar a aplicação das etapas do Processo de Enfermagem no cuidado à mulher na Atenção Primária à Saúde.

**Métodos:**

estudo transversal, desenvolvido de maio a julho de 2022, em 17 unidades básicas de quatro municípios brasileiros com 25 enfermeiros e 92 prontuários. Com os enfermeiros, aplicaram-se questionário e análise dos dados por estatística descritiva pelo *software* SPSS^®^, versão 25.0. Os prontuários foram lidos e analisados.

**Resultados:**

a execução do Processo de Enfermagem foi parcial, e todas as fases tinham registros incompletos. Avaliação e planejamento eram os mais presentes, e diagnóstico, implementação e evolução de enfermagem, os mais frágeis.

**Conclusões:**

a incompletude dos registros do Processo de Enfermagem compromete a documentação do trabalho da enfermagem sobre a clínica do paciente, podendo gerar implicações éticas e legais para os profissionais. É necessário investir em capacitações e despertar o interesse dos profissionais na aplicação e registro do Processo de Enfermagem.

## INTRODUÇÃO

A atuação do enfermeiro na Atenção Primária à Saúde (APS) no Brasil vem se consolidando como instrumento de mudanças nas práticas de atenção à saúde no Sistema Único de Saúde (SUS). O olhar tecnicista desse profissional foi substituído pela visão ampliada do cuidado, centrado na integralidade, na intervenção diante dos fatores de risco, na prevenção de doenças e na promoção da saúde^(1)^. Nesta conjuntura, o Processo de Enfermagem (PE) atua como ferramenta que possibilita a qualidade do cuidado. Desse modo, os enfermeiros têm a oportunidade de fortalecer a sua profissão e consolidar seu espaço profissional dentro da APS^(2)^.

Nesse âmbito do cuidado, os processos de trabalho em enfermagem são complexos e multifacetados, desdobrando-se classicamente em cinco processos: assistir; administrar; ensinar; pesquisar; e participar politicamente^(3)^. Para realizar seu trabalho com eficiência, o enfermeiro precisa dominar diversas habilidades, atuando em duas dimensões principais: assistencial e gerencial^(4)^. A dimensão assistencial tem como objeto o cuidado direto a indivíduos, famílias e comunidades, enquanto a dimensão gerencial foca na coordenação dos recursos e dos próprios agentes do cuidado para viabilizar a assistência. Portanto, espera-se que o enfermeiro na APS articule não apenas as práticas clínico-assistenciais, como consultas e solicitação de exames, mas também o gerenciamento da equipe e o monitoramento da situação de saúde da população^(3,5)^.

Para organizar e qualificar a dimensão assistencial, o PE se constitui como o principal instrumento metodológico, permitindo ao enfermeiro a tomada de decisão e o planejamento de ações de promoção, prevenção e intervenção^(3,6)^. Sendo assim, a aplicação do PE transcende a mera formalidade de registro; ela é a operacionalização do “processo de trabalho assistir”^(3)^, fundamentando o raciocínio clínico e a autonomia profissional no planejamento do cuidado integral na APS^(6)^.

No cenário brasileiro, o Conselho Federal de Enfermagem^(7)^, por meio da Resolução nº 736/2024, dispõe sobre a implementação do PE em todo o contexto socioambiental onde ocorre o cuidado de enfermagem, organizando-se em cinco etapas inter-relacionadas, interdependentes, recorrentes e cíclicas: avaliação de enfermagem; diagnóstico de enfermagem (DE); planejamento de enfermagem; implementação de enfermagem; e evolução de enfermagem^(7)^.

O PE é um método científico utilizado para planejar, executar e avaliar o trabalho do enfermeiro^(8)^. Trata-se de componente essencial das legislações que fornecem suporte legal à profissão da enfermagem, delineando as atividades privativas do enfermeiro. Sua efetivação favorece o trabalho desta categoria profissional, estabelecendo o cuidado ao paciente em etapas sequenciais, com intervenções baseadas em evidências científicas e pondo em prática o raciocínio lógico^(6)^.

Para embasar o PE, são utilizados sistemas de classificação, sendo os mais utilizados a Taxonomia da NANDA Internacional (NANDA-I)^(9)^, a Classificação das Intervenções de Enfermagem^(10)^, a Classificação dos Resultados de Enfermagem^(11)^, a Classificação Internacional para a Prática de Enfermagem^(12)^ e a Classificação Internacional das Práticas de Enfermagem em Saúde Coletiva^(13)^.

Destaca-se que o trabalho do enfermeiro, como agente fortalecedor da APS, deve proporcionar, por meio da consulta de enfermagem (CE) e embasada pelos sistemas de classificação, assistência de qualidade à população, com ações eficientes e aceitas pela comunidade^(14)^. Desse modo, é imprescindível que o enfermeiro busque continuamente o aprimoramento, visando à adequada determinação de intervenções na CE e reconhecendo que o PE é contínuo e permanente, o que reflete positivamente na qualidade da assistência^(15)^. Sabe-se que sua utilização é mandatória em todos os cenários em que esse profissional atua com indivíduos, famílias e comunidades. Nesse sentido, a APS se destaca como um importante espaço para essa implementação^(7,16,17)^.

Embora alguns estudos discorram sobre a aplicabilidade do PE na APS^(8,6,13,18)^, eles não contemplam as distintas regiões do Brasil em uma única pesquisa, nem são relacionados à área de saúde da mulher em sua totalidade. Nesse tocante, considerando que a área de saúde da mulher é aquela confere maior autonomia profissional ao enfermeiro, pela realização de atendimentos que envolvem coleta do exame de papanicolau, pré-natal e puerpério, queixas ginecológicas, eventos agudos, planejamento familiar, solicitação de exames e prescrição de medicamentos^(19)^, entre outras atividades que são caracterizadas como práticas avançadas de enfermagem^(20)^, este estudo apresenta o seguinte questionamento: os enfermeiros atuantes na APS aplicam o PE durante a CE em saúde da mulher?

## OBJETIVOS

Analisar a aplicação das etapas do PE no cuidado à mulher na APS.

## MÉTODOS

### Aspectos éticos

O estudo foi conduzido de acordo com as diretrizes éticas nacionais e internacionais, sendo aprovado pelo Comitê de Ética em Pesquisa do Hospital Israelita Albert Einstein - São Paulo, cujo parecer está anexado à presente submissão. Nesse sentido, este estudo atende às exigências fundamentadas na Resolução n^o^ 466/ 2012^(21)^ do Conselho Nacional de Saúde, que regulamenta pesquisas envolvendo seres humanos.

### Desenho, período e local do estudo

Trata-se de pesquisa observacional, multicêntrica, exploratória, de caráter transversal e abordagem quantitativa, norteado pela ferramenta *STrengthening the Reporting of OBservational studies in Epidemiology*, realizada em 17 Unidades Básicas de Saúde (UBSs) localizadas em quatro municípios brasileiros, nomeadamente Parelhas (Rio Grande do Norte, São Paulo (São Paulo), Manaus (Amazonas) e Carneiros (Alagoas), englobando as regiões Nordeste, Norte e Sudeste do Brasil. A escolha dos municípios se deu por conveniência, uma vez que as pesquisadoras residem nos estados mencionados, o que viabilizou a coleta de dados. O processo de seleção das UBSs também ocorreu por conveniência, após realização de convite por parte das pesquisadoras e aceite dos enfermeiros atuantes nesses cenários. A amostra incluiu enfermeiros que atuam na APS e em pleno exercício de suas funções no dia da coleta de dados, totalizando 25 participantes. Enfermeiros gestores foram excluídos do estudo.

### Protocolo de estudo

A coleta de dados ocorreu entre maio e julho de 2022. Na ocasião, as pesquisadoras abordaram e convidaram as participantes, explicando os objetivos, a metodologia, os riscos e os benefícios da pesquisa. Após a aceitação, os participantes assinaram o Termo de Consentimento Livre e Esclarecido e o Termo de Assentimento Livre e Esclarecido, quando necessário. Posteriormente, foi aplicado um questionário de caracterização específico para esse grupo populacional, utilizando a plataforma *Research Electronic Data Capture* (REDCap), além de um Termo de Autorização de Uso de Imagem e Voz.

Na sequência, foram analisados os prontuários e acessados os registros dos atendimentos realizados pelos enfermeiros, com objetivo de identificar o cumprimento das etapas do PE nas consultas. Os dados foram coletados por meio de cópias das evoluções em prontuários físicos ou eletrônicos, de acordo com o que a unidade dispunha, cedidos pelos enfermeiros participantes e arquivados em *drive* institucional, sendo analisados após finalização da coleta, no período entre agosto a setembro de 2022. No total, foram analisados 92 prontuários.

O instrumento de coleta é um *checklist* elaborado pelas pesquisadoras. Para tanto, foram adaptadas as etapas para o método Subjetivo, Objetivo, Avaliação e Plano (SOAP), considerando que este é o formato de registro disponível no prontuário nacional e-SUS^(22)^, sendo a aplicação realizada por meio da plataforma REDCap (Quadro 1).

**Quadro 1:** Instrumento de avaliação do Processo de Enfermagem por meio do método Subjetivo, Objetivo, Avaliação e Plano

ETAPAS PROCESSO DE ENFERMAGEM	ANÁLISE
Coleta de dados	
Dados subjetivos	O enfermeiro buscou compreender a demanda sob a ótica e perspectiva da usuária? A qualidade do processo envolveu a avaliação individual e global para identificar os fenômenos de enfermagem relevantes?
Dados objetivos	
Avaliação	
Exame físico	Inclui as necessidades da usuária, os problemas e recursos pessoais? Inclui o motivo que levou a usuária à consulta de enfermagem? Estavam descritas as situações social, de gênero, espiritual e aspectos fisiológicos?
Diagnóstico de enfermagem	
Apresentou diagnóstico de enfermagem?	Os diagnósticos de enfermagem estão registrados? Os diagnósticos de enfermagem são numerados em ordem para serem monitorados nos registros de enfermagem? Os diagnósticos de enfermagem contêm etiologias relacionadas aos sinais e sintomas correspondentes? Os diagnósticos de enfermagem levantados estão registrados de acordo com a taxonomia utilizado pela instituição? Se não, qual o enfermeiro utilizou?
Diagnóstico de enfermagem tem correlação com a coleta de dados apresentada?	
Utilizou CIAP?	
O CIAP tem correlação com a coleta de dados apresentada?	
Planejamento	
Metas/resultados esperados	O planejamento de enfermagem está registrado? Há uma linguagem padronizada utilizada? O planejamento de enfermagem está elaborado de acordo: como será realizado? Com que frequência? Por quem será realizado? O planejamento de enfermagem está registrado de acordo aos problemas ou diagnósticos levantados?

*CIAP - Classificação Internacional de Assistência Primária.*

*Fonte: Cordeiro^(20)^; Barreto^(23)^; Carrer^(24)^; Serranegra^(25)^; Reis^(26)^; Almeida^(27)^ - Recife, Pernambuco, Brasil, 2025.*

### Análise dos resultados e estatística

Durante a análise dos registros, atribuíram-se os respectivos valores: 0 = não se aplica; 1 = ausente (quando não há o registro de cada subitem em prontuário); 2 = parcialmente presente (quando há o registro de apenas um aspecto); e 3 = presente (quando o registro está completo).

Os dados foram transferidos para uma planilha eletrônica, e as variáveis foram analisadas segundo a estatística descritiva (frequência e porcentagem) utilizando-se o *software Statistical Package for the Social Sciences*, versão 25.0. Realizou-se avaliação dos dados, considerando-se completude, valores ilógicos e preenchimento dos dados. Foram calculados a média e desvio padrão das variáveis tempo de consulta e idade.

## RESULTADOS

Participaram deste estudo 25 enfermeiros, com predominância do sexo feminino (80%), cuja média de idade foi de 39,13± 7,36 anos. A maioria se identificou como de cor parda (60%), apresentando uma faixa salarial superior a sete salários mínimos (56%) e residindo na cidade de Manaus (40%). Na análise descritiva sobre a formação acadêmica, a maioria dos entrevistados relatou ter recebido aulas sobre o PE durante a graduação (88%), e 100% afirmaram ter formação em nível de pós-graduação, com 44% dessa formação voltados à especialização em saúde da família e comunidade, saúde coletiva ou saúde pública. A maior parte também participou de cursos na área de saúde da mulher no último ano (64%).

Quanto à experiência profissional, uma parcela expressiva dos enfermeiros afirmou ter entre seis e dez anos de atuação (48%) e de um a cinco anos de experiência na Estratégia Saúde da Família (36%), que era o tipo de unidade de saúde em que estavam inseridos durante a pesquisa (64%).

Na Tabela 1, está apresentada a distribuição das variáveis contextuais em valores absolutos e relativos dos enfermeiros participantes da pesquisa. De acordo com o exposto na tabela, em relação às condições de trabalho, a maioria dos enfermeiros tinha um tempo de deslocamento de até 30 minutos para chegar ao serviço, trabalhava 40 horas semanais, utilizava a taxonomia NANDA Internacional para elaborar DEs e aplicava a Teoria das Necessidades Humanas de Wanda Horta. A maioria não possuía outro vínculo empregatício, contava com uma equipe multiprofissional integrada à equipe de saúde, trabalhava com prontuário eletrônico e tinha uma sala dedicada às CEs. Uma parte considerável utilizava protocolos de enfermagem em sua rotina de trabalho (56%), sendo os mais citados os Cadernos da Atenção Básica e os protocolos do Ministério da Saúde. Em relação à motivação para o exercício da profissão, uma parcela expressiva expressou satisfação (52%) (Tabela 1).

**Tabela 1:** Distribuição das variáveis contextuais em valores absolutos e relativos dos enfermeiros participantes da pesquisa (N=25), Recife, Pernambuco, Brasil, 2025

Variáveis	Categorias	n	%
Tempo gasto para se deslocar ao trabalho	Até 30 minutos	12	48,0
	Entre 31 minutos e 60 minutos	8	32,0
	Entre 61 minutos e 120 minutos	4	16,0
	Mais de 121 minutos	1	4,0
Carga horária de trabalho semanal	30 horas semanais	1	4,0
	40 horas semanais	22	88,0
	Outra	2	8,0
	NANDA-I	8	32,0
Qual taxonomia de diagnóstico de enfermagem você utiliza em seu cotidiano de trabalho?	Nenhuma	8	32,0
	Classificação Internacional para a Prática de Enfermagem	6	24,0
	Outros	3	12,0
Qual teoria de enfermagem você utiliza em seu cotidiano de trabalho?	Teoria das Necessidades Humanas - Wanda Horta	13	52,0
	Não utiliza teoria de enfermagem	7	28,0
	Outros	3	12,0
	Teoria Ambientalista - Florence Nightingale	1	4,0
	Teoria do Autocuidado - Dorothea Orem	1	4,0
Você possui outro vínculo empregatício fora da Atenção Primária à Saúde?	Não	15	60,0
	Sim	10	40,0
Você possui uma equipe multiprofissional vinculada à sua equipe de saúde?	Sim	16	64,0
	Não	9	36,0
Há prontuário eletrônico na sua unidade?	Sim	22	88,0
	Não	3	12,0
Sua unidade dispõe de sala para consulta de enfermagem?	Sim	18	72,0
	Não	7	28,0
Na sua unidade, são adotados protocolos de enfermagem?	Sim	14	56,0
	Não	11	44,0
	Caderno de Atenção Básica do Ministério da saúde	20	80,0
	Protocolos do Ministério da Saúde	18	72,0
	Protocolo da sua secretaria municipal de saúde	11	44,0
	Protocolo do Conselho Regional de Enfermagem	7	28,0
	Protocolo estadual	3	12,0
	Protocolo de outro município	2	8,0
O quanto motivado você se sente para exercer a sua função profissional?	Muito satisfeito	5	20,0
	Satisfeito	13	52,0
	Um pouco satisfeito	3	12,0
	Nem satisfeito nem insatisfeito	1	4,0
	Insatisfeito	3	12,0

*Nota: sinais convencionais utilizados: N - número de participantes; % - percentual representativo.*

Quanto ao tipo de CE, 28% das usuárias procuraram atendimento pré-natal, seguidas por 26%, que buscaram a realização do exame de Papanicolau, e 10%, que compareceram para apresentar resultados de exames. Entre os principais sintomas relatados pelas pacientes durante a coleta de dados, parcela significativa mencionou dificuldades para dormir (26%), tontura (22%), fraqueza (20%), episódios de choro ou tristeza (16%), e sensação de falta de ar (13%).

O tempo médio de consulta foi de 22,88±17,17 minutos, sendo que a maioria alcançou tempo ≤ a 20 minutos (55%). Houve casos em que a usuária estava acompanhada durante a consulta (20%), sendo os acompanhantes filhos (6%) e irmãos (6%). Em 42% das CEs, houve interrupções, e em 64%, o enfermeiro procurou o médico para realizar interconsulta. Entre as consultas, 32% tratavam-se de retorno ou seguimento longitudinal, enquanto em 67%, o enfermeiro mencionou a necessidade de um novo encontro. O registro das CEs esteve presente em 92% dos prontuários, sendo 73% em prontuário eletrônico e no formato SOAP.

Tratando-se das etapas do PE, a avaliação de enfermagem foi registrada, em maioria, de forma parcial, com predominância de dados subjetivos (49%), objetivos (74%) e exame físico (41%). Os DEs estavam ausentes em 71% dos prontuários analisados e, quando presentes, somente 9% tinham relação com a coleta de dados. O formato do registro se deu por intermédio da Classificação Internacional de Atenção Primária (CIAP) (74%), e 62% estavam presentes na relação do CIAP com a coleta de dados.

Em referência à etapa do planejamento, verificou-se que 14% dos registros apresentavam a descrição das metas e dos resultados esperados de forma parcial, e 4% apresentavam relação com o DE. Outrossim, a etapa de prescrição do plano apresentou percentual de 61% de registros em prontuários, sendo que 11% dessas prescrições são aplicadas aos resultados esperados e ao diagnóstico (10%), ambas parcialmente. A etapa de implementação esteve presente em somente 5%, e a evolução do plano foi evidenciada em 12% dos prontuários.

## DISCUSSÃO

Este estudo evidenciou que os enfermeiros executavam as CEs de maneira incipiente e frágil, e aplicavam as etapas do PE de forma parcial, com registro incompleto, sendo a avaliação e o planejamento de enfermagem as etapas que mais se fizeram presentes, enquanto que a implementação e a evolução do plano foram as mais incipientes. Estudos apresentaram resultados semelhantes, quando se referem à fragmentação na realização da CE, à fragilidade nos registros^(28-30)^ e ao desenvolvimento parcial das etapas do PE na APS^(23-27)^.

A fragmentação do PE é percebida frequentemente no preenchimento de prontuários na APS, e são identificados diversos fatores que dificultam a aplicabilidade do PE, sendo os mais citados pela literatura: inexistência de capacitação para aplicação do PE e de espaços físicos para realizar a CE; sobrecarga de trabalho; desvalorização profissional^(6,31,32)^; dimensionamento inadequado da equipe; ausência de apoio institucional e da equipe multiprofissional; interrupções durante a CE; limitações acerca dos conhecimentos científicos; percepção de desperdício de tempo na execução do PE^(29,32)^; e inexistência de protocolos que subsidiem sua aplicabilidade na prática^(8,33)^.

Os enfermeiros compreendem que o conhecimento sobre PE é essencial para sua implementação e documentação. No entanto, ao mesmo tempo, eles consideram que esse conhecimento é insuficiente desde a formação acadêmica, o que resulta em um uso inadequado e parcial dessa tecnologia^(18,32)^. Embora a maior parte dos participantes deste estudo tenha recebido aulas sobre o PE durante a graduação, permanecem os vestígios da formação que evidenciam essa limitação na atuação na APS. Compreender essa realidade é essencial para a elaboração de práticas inovadoras, através de iniciativas que unam o ensino e a prática profissional^(34)^.

A falta de suporte por parte dos órgãos de fiscalização e dos gestores na APS também tem sido apontada como uma fragilidade frequente que impacta negativamente a implementação do PE^(29,30,32)^. Destarte, reforça-se a importância da implementação de estratégias que assegurem a efetiva operacionalização do PE nos serviços de saúde, considerando as especificidades das transformações tecnológicas, o avanço conceitual e o desenvolvimento do exercício e da regulamentação da profissão da enfermagem no Brasil^(35,36)^.

Sob esse enfoque, o papel das universidades neste cenário é mediar e se voltar ao fortalecimento das bases teóricas com que tange ao PE, contribuindo para o raciocínio clínico e destacando que a CE em saúde da mulher não está relacionada apenas às queixas ginecológicas, mas à integralidade do cuidado, considerando aspectos biopsicossociais, culturais e individuais. Portanto, o enfermeiro precisa prestar assistência de qualidade com base nas melhores evidências científicas, aplicando o PE em todas as consultas^(23)^.

Para tanto, observa-se a necessidade de reorientação dos currículos das escolas de enfermagem, no qual o conteúdo referente ao PE na APS seja inserido e ofertado de maneira específica e coordenada. A utilização deste na formação acadêmica proporciona ao aluno aproximação com a legislação profissional, favorecendo a visão ampliada das ações de enfermagem^(27)^. Concomitantemente, os gestores locais e as instâncias maiores precisam se sensibilizar, fortalecendo e promovendo oficinas de educação permanente sobre o PE e a ampliação do escopo da enfermagem, possibilitando que o enfermeiro incorpore as competências esperadas durante as consultas^(23)^.

Outrossim, convém destacar o papel do enfermeiro enquanto sujeito ativo neste processo. É impreterível que este profissional atue na busca do conhecimento para qualificação e aperfeiçoamento do cuidado prestado ao paciente, assim como execute o que é da autonomia e competência dele.

Compreende-se que a autonomia está relacionada ao conhecimento profissional, pois os enfermeiros que se capacitam apresentam melhor domínio do trabalho, contribuindo para prestação da assistência qualificada e adquirindo visibilidade que, consequentemente, refletirá na autonomia conquistada^(37)^. Nessa perspectiva, para que se possa prestar atendimento autônomo, é preciso reconhecer os conhecimentos específicos da enfermagem. A ocorrência de falhas nesse processo dificulta a realização do raciocínio clínico, podendo tornar o PE uma mera execução de tarefas assistenciais, o que o descaracteriza como um método científico^(38)^.

Neste estudo, a avaliação de enfermagem e a prescrição do plano foram as etapas mais registradas pelos enfermeiros, corroborando outras pesquisas^(28,34)^. Isto pode ser justificado pelo fato de o enfermeiro dispor de manuais, Cadernos da Atenção Básica, protocolos ministeriais e protocolos institucionais que embasam sua prática profissional, contribuindo para o exercício das atividades de enfermagem de forma autônoma^(19)^. A utilização desses documentos pode contribuir de forma significativa como subsídio para a implementação do PE na APS^(6)^.

Em contrapartida, a implementação e evolução foram evidenciadas como as etapas mais incipientes, fragilizando a integralidade e completude do PE pelo profissional que prescreveu os cuidados. Segundo Faruch *et al*.^(39)^, a implementação do PE tem como objetivo garantir, com base em evidências científicas, a melhor conduta e a qualidade da assistência prestada aos pacientes. Isso contribui para o desenvolvimento contínuo das competências teóricas, práticas e interativas dos profissionais de saúde.

Quando o enfermeiro incorpora o PE em sua prática, ele assegura que suas atividades sejam realizadas de maneira eficiente e embasadas em evidências científicas. Isso proporciona a ele e a toda a sua equipe multiprofissional a segurança necessária para a execução dos procedimentos. Dessa forma, são oferecidos recursos para o planejamento do cuidado em resposta à prescrição de enfermagem, organizando e orientando a equipe em relação às prioridades dos atendimentos, o que favorece uma abordagem integral e holística aos pacientes^(40)^.

Tratando-se da teoria de enfermagem utilizada em seu ambiente de trabalho, os participantes relataram o referencial da Teoria das Necessidades Humanas Básicas de Wanda Horta, precursora do PE no Brasil, que orienta a avaliação de enfermagem e sustenta o registro das demais etapas do processo. Essa teoria aponta a CE como fator essencial para melhorar a qualidade do cuidado, tornando-o mais humanizado e permitindo ao enfermeiro atuar com maior autonomia e independência diante do paciente^(8)^.

Cabe ressaltar que o desenvolvimento completo do PE deve ser sustentado por uma teoria de enfermagem e estar associado ao registro adequado no prontuário do paciente^(41)^, posto que, quando bem documentado, permite o cuidado centrado no paciente, refletindo a qualidade da assistência prestada^(42)^.

A aparente contradição entre a elevada autonomia percebida pelos enfermeiros no cuidado à saúde da mulher^(43)^ e a aplicação apenas parcial do PE revela a complexidade do processo de trabalho na APS, que engloba não apenas o “assistir”, mas também o “administrar”, o “ensinar” e o “participar politicamente”^(3)^. Sugere-se, assim, que a autonomia relatada pode estar sendo exercida mais na execução de protocolos ministeriais, como pré-natal^(5)^ e rastreamento de câncer^(43)^, do que na aplicação plena do PE.

Este estudo corrobora que a assistência à saúde da mulher, embora prioritária na agenda, foca-se intensamente no ciclo reprodutivo, negligenciando áreas como o climatério ou o enfrentamento da violência^(43)^. A fragilidade encontrada nas etapas “implementação” e “evolução” do PE é um ponto crítico, pois são elas que garantem a continuidade e reavaliação do cuidado. A adoção parcial transforma o PE em um ato fragmentado e burocrático, limitando seu potencial. Isso é particularmente relevante, pois evidências indicam que é a qualidade do processo de cuidado (que o PE deveria garantir) que impacta positivamente os resultados de saúde, sendo capaz, inclusive, de minimizar os efeitos de iniquidades socioeconômicas^(44)^.

Portanto, a falha em aplicar integralmente o PE, mesmo em um cenário de autonomia, representa uma subutilização dessa mesma autonomia profissional, que deveria ser o pilar para a qualificação da assistência. Dessa forma, é imprescindível não naturalizar a utilização parcial do PE, em especial na área de saúde da mulher. Além disso, ao abrir mão da utilização do PE, a enfermagem presta um desserviço à própria categoria, secundarizando o corpo de conhecimento que lhes confere embasamento científico, contribui para evolução da profissão e garante o poder de decidir sobre sua conduta na assistência ao paciente^(45)^.

### Limitações do estudo

A limitação deste estudo pode estar relacionada ao número amostral, porém, como foram estudadas diferentes regiões do Brasil, foi mantida a heterogeneidade da amostra nos cenários pesquisados. Destaca-se que esta pesquisa possibilitou compreender a importância e necessidade do desenvolvimento da CE de maneira sistematizada e resolutiva e da aplicabilidade do PE em sua completude.

### Contribuições para a área da saúde, enfermagem ou políticas públicas

A adoção de ferramentas específicas para a atuação dos enfermeiros aprimora a qualidade do trabalho profissional, fortalece o cuidado sistematizado, estrutura os processos e as rotinas de trabalho, além de facilitar o registro e a organização das informações^(23,24,32)^. Diante do exposto, reafirma-se a necessidade de o enfermeiro se empoderar daquilo que lhe é privativo, buscando conhecimento para o aumento da valorização profissional e a realização de intervenções baseadas em evidências^(46,47)^. Este estudo contribui para a reflexão do enfermeiro acerca da importância da implementação do PE como estratégia para o gerenciamento do cuidado e consolidação da autonomia profissional.

## CONCLUSÕES

A incompletude dos registros do PE compromete a documentação do trabalho da enfermagem sobre a clínica do paciente, podendo gerar implicações éticas e legais para os profissionais. Nesse tocante, é impreterível o investimento em capacitações, educação permanente e continuada sobre o PE na consulta à saúde da mulher voltado aos enfermeiros que atuam na APS, bem como o interesse por parte desses profissionais em aplicá-lo na prática assistencial.

A não realização do PE, conforme os preceitos estabelecidos pela Resolução n^o^ 736/2024^(7)^, pode ocasionar impactos adversos na qualidade da assistência, incluindo a falta de avaliação sistemática do paciente, comprometendo a continuidade do cuidado, a identificação precoce de complicações e a personalização das intervenções, o que resulta em maior risco à segurança do paciente e em desfechos clínicos indesejados.

Este estudo promove reflexões acerca dos papéis do profissional de enfermagem e vislumbra o potencial da CE como instrumento metodológico para sistematizar a assistência e fortalecer a APS.

## Data Availability

Os dados de pesquisa só estão disponíveis mediante solicitação.
